# Neuroactive steroid effects on autophagy in a human embryonic kidney 293 (HEK) cell model

**DOI:** 10.1038/s41598-024-51582-x

**Published:** 2024-01-10

**Authors:** Sofia V. Salvatore, Ma. Xenia G. Ilagan, Hongjin Shu, Peter M. Lambert, Ann Benz, Mingxing Qian, Douglas F. Covey, Charles F. Zorumski, Steven Mennerick

**Affiliations:** 1 Departments of Psychiatry, Washington University in St. Louis School of Medicine, 660 S. Euclid Ave., MSC 8134-0181-0G, St. Louis, MO 63110 USA; 2 Developmental Biology, Washington University in St. Louis School of Medicine, 660 S. Euclid Ave., MSC 8134-0181-0G, St. Louis, MO 63110 USA; 3 High-Throughput Screening Core, Center for Drug Discovery, Washington University in St. Louis School of Medicine, 660 S. Euclid Ave., MSC 8134-0181-0G, St. Louis, MO 63110 USA; 4 Medical Scientist Training Program, Washington University in St. Louis School of Medicine, 660 S. Euclid Ave., MSC 8134-0181-0G, St. Louis, MO 63110 USA; 5 Taylor Family Institute for Innovative Psychiatric Research, Washington University in St. Louis School of Medicine, 660 S. Euclid Ave., MSC 8134-0181-0G, St. Louis, MO 63110 USA

**Keywords:** Macroautophagy, Depression, Cellular neuroscience, Pharmacodynamics

## Abstract

Neuropsychiatric and neurodegenerative disorders are correlated with cellular stress. Macroautophagy (autophagy) may represent an important protective pathway to maintain cellular homeostasis and functionality, as it targets cytoplasmic components to lysosomes for degradation and recycling. Given recent evidence that some novel psychiatric treatments, such as the neuroactive steroid (NAS) allopregnanolone (AlloP, brexanolone), may induce autophagy, we stably transfected human embryonic kidney 293 (HEK) cells with a ratiometric fluorescent probe to assay NAS effects on autophagy. We hypothesized that NAS may modulate autophagy in part by the ability of uncharged NAS to readily permeate membranes. Microscopy revealed a weak effect of AlloP on autophagic flux compared with the positive control treatment of Torin1. In high-throughput microplate experiments, we found that autophagy induction was more robust in early passages of HEK cells. Despite limiting studies to early passages for maximum sensitivity, a range of NAS structures failed to reliably induce autophagy or interact with Torin1 or starvation effects. To probe NAS in a system where AlloP effects have been shown previously, we surveyed astrocytes and again saw minimal autophagy induction by AlloP. Combined with other published results, our results suggest that NAS may modulate autophagy in a cell-specific or context-specific manner. Although there is merit to cell lines as a screening tool, future studies may require assaying NAS in cells from brain regions involved in neuropsychiatric disorders.

## Introduction

Major depressive disorder is a debilitating and chronic illness with unknown etiology and pathophysiology^1^. Due to this lack of mechanistic understanding, current antidepressant treatments, in particular monoamine pharmacological approaches, have inconsistent responses and take weeks to exhibit clinical effects. There is an urgent need for the development of new, more rapidly acting antidepressants with fewer side effects. Neuroactive steroids (NAS) may be rising to fill this role, as the FDA-approved medication brexanolone, a formulation of the natural neurosteroid allopregnanolone (AlloP), persistently reduces depressive symptoms in women with postpartum depression upon a 60 h infusion^2,3^. Moreover, synthetic AlloP analogues currently in clinical trials may be useful for major depressive disorder^4,5^. However, the mechanisms driving these rapid and durable therapeutic effects are not well understood.

Macroautophagy (autophagy), a degradation pathway that is essential for preserving cellular homeostasis, may participate in the antidepressant effects of NAS. Dysregulated autophagy has been implicated in clinical depression, with multiple antidepressant drugs affecting this process^6^. First, AlloP, a positive allosteric modulator of GABA-A receptors, increases autophagy^7^. In addition, two conventional antidepressants (amitriptyline and fluoxetine) spur autophagy, linked causally to antidepressant benefit^8^. Autophagy can directly affect synaptic function and intrinsic excitability^9,10^, offering direct substrates to affect neuronal communication. Moreover, autophagy itself is modulated by synaptic activity^11^. Finally, a human clinical study found that rapamycin (an autophagy inducer) prolonged the antidepressant effects of ketamine, suggesting an important role for autophagy in therapeutic outcomes^12^.

The best known target of NAS are GABA-A receptors, and structure–activity relationships for NAS on GABA-A receptors have been well studied^13^. However, structural and cellular requirements for autophagy remain mostly unexplored. Autophagy induction by AlloP in the rat retina fails to exhibit enantioselectivity^14^, suggesting that NAS may act through unconventional pathways distinct from GABA-A receptor modulation. Both amitriptyline, a tricyclic, and fluoxetine, a selective serotonin reuptake inhibitor (SSRI), induce autophagy^8^ but share very little structural overlap. A common denominator may be lysosome, Golgi membrane, and ER interference. Uncharged NAS are known to accumulate in somatic Golgi of neurons^15^, but likely also permeate lysosomes and the ER, targets relevant to autophagy, as a result of their hydrophobicity. We hypothesized that uncharged NAS, but not charged NAS, increase autophagy because of differential lysosome penetration. In addition, we predicted that uncharged NAS will not exhibit enantioselectivity if effects are driven strictly by lysosome accumulation and effects on membranes, rather than effects on chiral protein targets.

Here, we examined the modulation of autophagy in response to structurally diverse NAS analogues using a stably expressed, ratiometric fluorescent probe, pcDNA3-GFP-LC3-RFP-LC3Δ3^16^ in human embryonic kidney 293 (HEK) cells. We quantified autophagy using microscopy and high-throughput microplate formats. We also expressed the probe in primary astrocytes. The adopted probe is not reliant on lysosomal inhibitors, and the RFP variant serves as an internal control for probe expression^16^.

Contrary to expectations and previous studies, we find that NAS do not strongly induce autophagy in HEK cells or astrocytes, even when tested in combination with known autophagy inducers (Torin1 and starvation). We conclude that NAS effects on autophagy are context-dependent and that this needs to be accounted for in screening assays.

## Methods

### Human embryonic kidney 293 (HEK) cell culture

HEK cells (ATCC CRL-1573) were stably transfected with 0.5 µg pcDNA3-GFP-LC3-RFP-LC3ΔG^16^ using Lipofectamine 2000 (Invitrogen, 11668030) for 4 h at 37 °C with 5% CO_2_ in Opti-MEM (Gibco, 31985070) _._ Following incubation, the medium was exchanged for serum-containing medium. Cells were selected with 750 µg of G418 and grown in Dulbecco’s Modified Eagle’s medium (DMEM) 1 g/L glucose (Gibco, 11885084) with 10% heat-inactivated fetal bovine serum (FBS) (Gibco, 16140063), and 1% GlutaMAX (Gibco, 35050061). Cells were maintained with 400 µg of G418 in media at 37 °C with 5% CO_2_.

### Primary cortical astrocyte culture

All procedures were carried out in accordance with National Institute of Health (NIH) guidelines and approved by the Washington University Institutional Animal Care and Use Committee, protocol 22-0344. Procedures involving animals are reported in accordance with ARRIVE guidelines (https://arriveguidelines.org). Primary cortical astrocyte cultures were prepared from postnatal day 4–7 Sprague Dawley rat pups of either sex. Mouse pups were anesthetized with halothane and rapidly decapitated. Brains were carefully stripped of their meninges and digested with 1 mg/mL papain for 20 min at 37 °C with 5% CO_2_, then rinsed with 5–5 media (Minimum Essential medium (Gibco, 11090081) supplemented with heat-inactivated horse serum (5%) (Gibco, 26050070), fetal bovine serum (5%), 17 mM glucose, 400 µM glutamine, 50 µg/ml streptomycin, and 50 U/ml penicillin). The tissue was then triturated and plated into T-25 flasks which were pre-coated with 0.1 mg/mL poly-D-lysine hydrobromide (PDL) (Sigma-Aldrich, P7280). Astrocytes were kept at 37 °C with 5% CO_2_. The following day, media was aspirated and fresh 5–5 was added. Media was changed once again 3 days after plating and 7 days after plating, with the addition of 20 µL (6.7 µm) of cytosine beta-D-arabinofuranoside (AraC). After, media was changed once a week and AraC was added once every other week, until astrocytes reached around 20–30 days in vitro (DIV).

Astrocytes were plated in 35 mm dishes pre-coated with PDL for transfection with 0.5 µg of pcDNA3-GFP-LC3-RFP-LC3ΔG, using Lipofectamine 2000. The transfection protocol consisted of 4 h incubation at 37 °C in Neurobasal medium (Gibco, 21103049) containing plasmids, 25 µM D-APV, 1 µM NBQX, and Lipofectamine 2000. Following the incubation, the medium was exchanged for 5–5.

### Treatments

Cells were treated with drugs or starvation media Earl’s Balanced Salt Solution (EBSS) (Thermo Fisher, 24010043) 24 h after plating in 35 mm dishes or 96-well plates (50% confluence) for 24 h (unless specified) in an incubator at 37 °C with 5% CO_2_. For astrocyte experiments, cells were treated 24 h after transfection under the same conditions described above. Drug treatments were at 1:1000 dilutions, using 0.1% DMSO as a vehicle control, and media removal and replacement as a control for starvation.

Drugs used were: rapamycin (LC—Laboratories, R-5000 or Tocris, 1292), Torin1 (LC—Laboratories, T7887), Bafilomycin A_1_ (MilliporeSigma Calbiochem, 19600-010UG), non-commercial NAS were custom synthesized in house. We used NAS at 1 and 10 µM as having been shown effective in previous literature, including autophagy^7^ and GABA-A receptor modulation^13^. Note that 10 µM of GABA-A receptor modulating NAS is supraphysiological and near the solubility limit for uncharged NAS.

### Microscopy

Images were taken on a Nikon Eclipse TE2000-S microscope equipped with epi-fluorescence illumination and a camera (Photometrics, CoolSNAP ES^2^). Images were acquired with Micro-Manager 2.0 software. 10× images from 3 different fields were taken for each treatment. Oil-immersion 60× images were taken for GFP/RFP localization using cells that were plated on glass cover slips in a 35 mm dish. Following treatment, they were fixed for 10 min in 4% paraformaldehyde/0.02% glutaraldehyde in PBS at room temperature. Cells were washed 3 times with PBS and then cover slips were placed on slides and imaged. Image capture order: phase, GFP, RFP, Alexa Fluor 647 (when used).

### Image analysis

GFP and RFP fluorescence were quantified using the analyze particles tool in FIJI. 200–500 cells were analyzed from each dish for HEK cell experiments, while ~ 6 cells were analyzed from each dish for astrocyte experiments. Intensity values from the entire cell were taken from the RFP image, whose ROI was then copied onto the GFP image for intensity values. Both channels were background subtracted, followed by the calculation of GFP/RFP.

### High-throughput screening using a microplate reader

Stably transfected HEK cells < P10 were seeded on 96 well plates (Santa Cruz, sc-204468) coated with PDL at 17,000 cells/well. After 24 h, cells were treated and incubated for another 24 h (unless specified). Media was exchanged with HEPES-buffered live cell imaging solution (Invitrogen, A14291D), and cells were assayed using a microplate reader (Molecular Devices, Flexstation 3) with excitation/emission 488/509 nm GFP and 584/607 nm RFP. Every treatment (including controls) had 4 technical replicates (wells) in each experiment. Control values for each experiment were calculated by averaging the 4 technical replicates. Ratiometric values were expressed by normalizing replicates within a condition to the experiment’s mean control value (individual replicate/average controls in that experiment *100). Controls values exhibited little variability; in a representative 2 independent experiments (Fig. 2c) normalized individual values were 100 ± 3.5% (mean ± SEM, n = 8, 2 independent experiments).

### High-content imaging

Stably transfected HEK cells < P10 were seeded into 96 well plates (Santa Cruz, sc-204468) coated with PDL at 15,000 cells/well and incubated at 37 °C with 5% CO_2_. After 24 h, cells were treated and incubated with drugs for another 24 h. After treatment, Hoescht stain was added to media and left in incubator for 10 min. Media was exchanged with HEPES-buffered live cell imaging solution, and cells were imaged at 10× using the InCell 2000 Analyzer (GE Healthcare) with excitation/emission 490/525 nm GFP, 579/624 nm RFP, 350/455 nm Hoescht. Each well had 4 fields imaged. Every treatment (including controls) had 4 technical replicates (wells) in each experiment.

Images were analyzed using the Multi Target Analysis Module of the InCell Analyzer 1000 Workstation Software version 3.7). Individual cells (nuclei) were identified using the top-hat segmentation method in the Hoescht channel. A cytoplasmic sampling region was then established by dilating 2 µm from the nuclear region (i.e., collar segmentation). The average intensities in each cell were determined from the combined nuclear and cytoplasmic regions in both the GFP and RFP channels. GFP and RFP mean intensity from each cell were averaged for each well. Then, both channels were background subtracted, followed by the calculation of average GFP/RFP for each well. Around 15,000–18,000 cells were analyzed per well. Ratiometric values were expressed by normalizing replicates within a condition to the experiment’s mean control value (individual replicate/average controls in that experiment *100).

### Immunostaining

Following treatment, astrocytes were fixed for 10 min in 4% paraformaldehyde/0.02% glutaraldehyde in PBS at room temperature. Cells were washed 3 times with PBS, blocked in 10% normal goat serum in PBS with 0.1% Triton X-100 for 15 min, incubated in primary rabbit anti-GFAP antibody (Millipore, AB 5804) diluted 1:1000 in block solution at 4 °C, shaken overnight and washed 3 times with PBS. Subsequently, cells were incubated in secondary Alexa Fluor 647 conjugated goat anti-rabbit antibody (Invitrogen, A-21245) diluted 1:500 in PBS for 1 h at room temperature, in the dark with shaking. Finally, cells were washed 4 times with PBS and maintained in PBS for imaging.

### Statistical analysis and figure preparation

All image measurements were obtained from the raw data. GraphPad Prism was used to plot graphs and perform statistical analyses, including dose–response curves. Error bars represent mean ± standard error of mean (SEM). Each replicate was treated as an independent sample. For presentation of images, maximum and minimum gray values were adjusted in FIJI and assembled in InkScape.

## Results

### Autophagy is reliably detected by low passage HEK cells stably expressing pcDNA3-GFP-LC3-RFP-LC3ΔG

**Figure 1:**
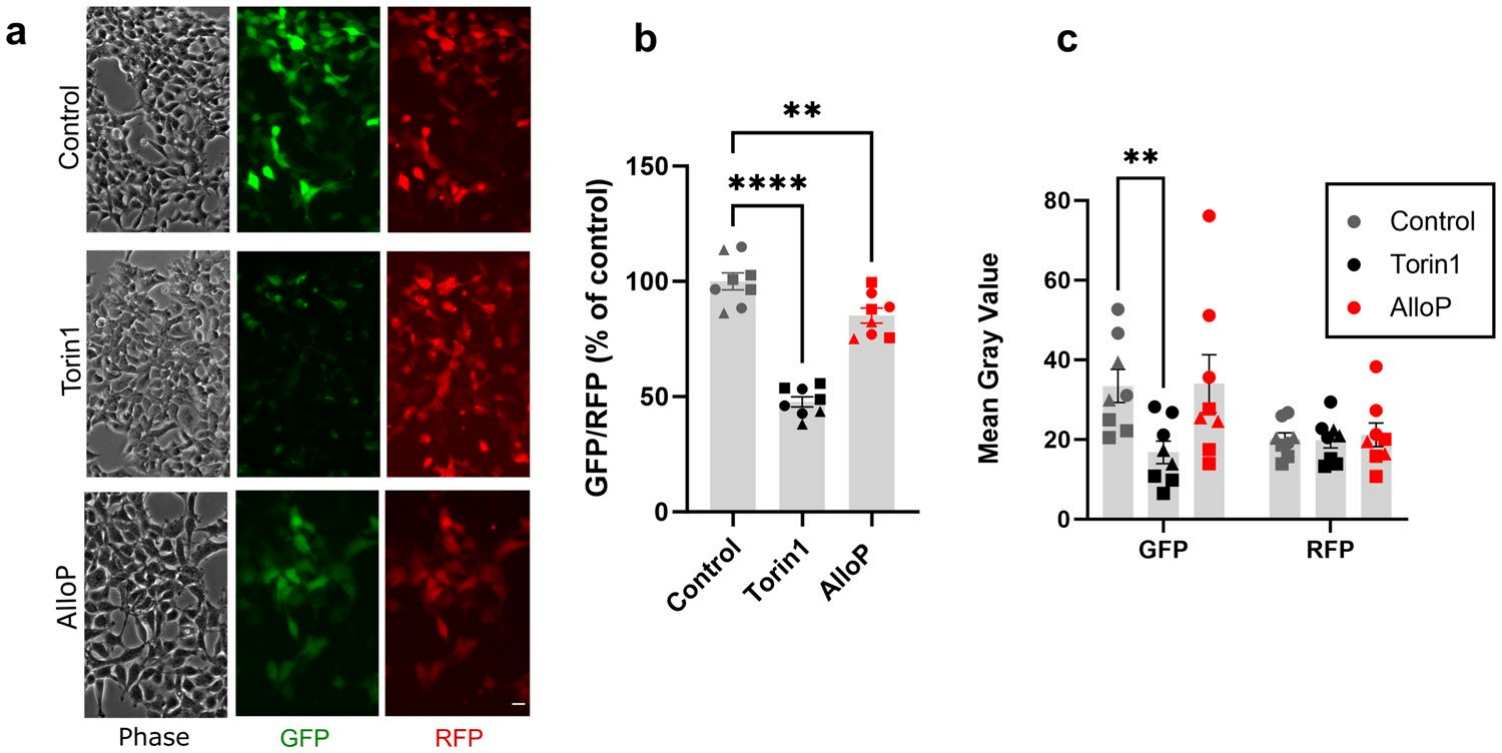
Autophagy is reliably detected by HEK cells stably expressing pcDNA3-GFP-LC3-RFP-LC3ΔG. (**a**) HEK cells in phase contrast, GFP, and RFP channels at 10× after a 24 h treatment with 500 nM Torin1 or 1 µM AlloP. Scale bar: 25 µm. (**b**) Background-subtracted mean fluorescence ratios relative to their vehicle-treated control. Each point represents a 35 mm dish (250–800 cells analyzed per dish) (n = 8 per group) from 3 independent experiments. Circles represent first experiment, squares second, and triangles third. A one-way ANOVA (*F* (2, 21) = 74.49, *p* < 0.0001) showed differences between treatments, and Dunnett’s multiple comparisons revealed a difference between control and Torin1 (****p < 0.0001) and between control and AlloP (**p = 0.0056). (**c**) Separate GFP and RFP mean fluorescence for each treatment, 3 independent experiments. A repeated measures two-way ANOVA revealed a difference of fluorescent protein (*F* (1, 21) = 16.25, *p* = 0.0006) and no difference of treatment (*F* (2, 21) = 2.077, *p* = 0.1503), with a significant interaction of treatment x fluorescent protein (*F* (2, 21) = 7.819, *p* = 0.0029). Sidak’s multiple comparisons revealed a difference between control and Torin1 GFP (**p = 0.0093).

To assay NAS effects on autophagy, we stably transfected HEK cells with pcDNA3-GFP-LC3-RFP-LC3ΔG^16^, a fluorescent ratiometric probe wherein GFP/RFP levels inversely correlate with autophagic flux. HEK cells were chosen because of their prominent role in studying neuronal ion channel function^17^ as well as other neuronal pathways, including autophagy^18,19^. GFP-LC3 is targeted for autophagic degradation through lysosomal quenching and protein degradation while RFP-LC3ΔG is an internal control that is not subject to autophagic degradation. High-magnification images of HEK cells transfected with the probe exhibited fluorescent puncta consistent with autolysosome formation (Supplementary Fig. 1). To confirm that the probe reliably detects pharmacological induction of autophagy, we treated cells with Torin1 (0.5 µM for 24 h), an mTOR inhibitor and potent autophagy inducer, and with allopregnanolone (AlloP; 1 µM for 24 h), a neurosteroid that induces autophagy in mammalian retina^7^. Torin1 strongly decreased the GFP/RFP ratio as expected (Fig. 1a–c), mainly by decreasing GFP fluorescence (Fig. 1a, c). RFP fluorescence remained constant relative to vehicle control (Fig. 1a, c). RFP-LC3ΔG does not bind to the autophagosome membrane, thus it can be either retained within an autophagosome or exist freely in the cytoplasm. GFP fluorescence proved too dim to allow assessment of cellular distribution following Torin1 treatment. Compared with the effect of Torin1, 24 h treatment with 1 µM AlloP, a concentration believed to represent a therapeutic level^20^, modestly reduced the GFP/RFP ratio, although this was not clearly associated with GFP quenching (Fig. 1b, c). Overall, the results suggest marginal AlloP effects on autophagic flux.

**Figure 2:**
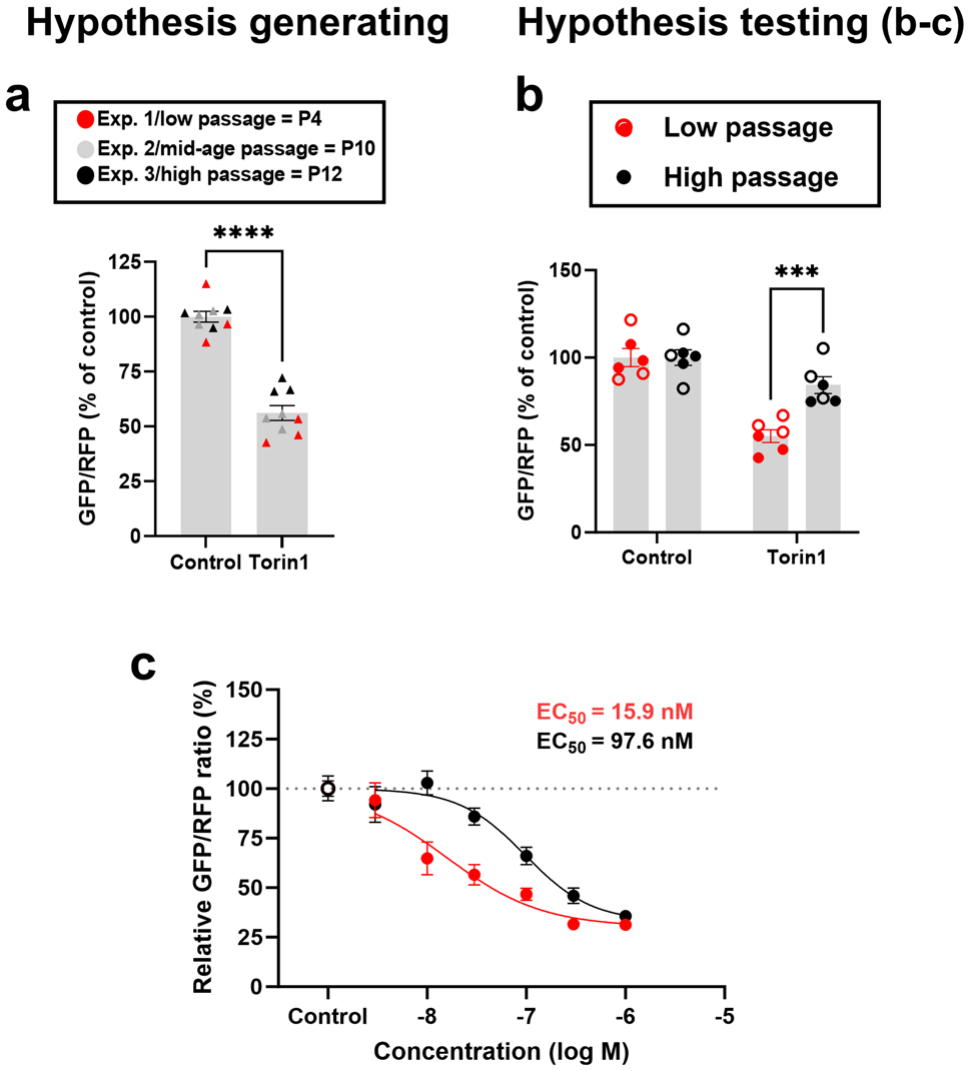
Passaging impairs pharmacological autophagy induction. (**a**) 500 nM Torin1 effect weakened with increasing cell passages. Overall, Torin1 reduced fluorescence ratio relative to control, unpaired *t*-test: *t*(16) = 10.54, ****p < 0.0001, n = 3 per condition in 3 replicates. 6 dishes from the hypothesis-generating studies in panel A were also those used for quantification of control and Torin1 in Fig. 1. (**b**) In independent microscopy experiments, we directly compared Torin1 effect in P5 + P7 versus P18 + P20 HEK cells to verify the observation (n = 3 per condition in 2 independent replicates, closed symbols first experiment open second). The Torin1 effect was impaired in higher passage cells compared to lower passage (***p = 0.0004, two-way ANOVA with Sidak’s multiple comparisons). The two-way ANOVA also revealed a significant effect of treatment (*F* (1, 20) = 44.29, *p* < 0.0001) and of passage number (*F* (1, 20) = 10.28, *p* = 0.0044), with a significant interaction of treatment x passage number (*F* (1, 20) = 10.28, *p* = 0.0044). (**c**) Dose–response curves of Torin1 for low (P6 + P9) and high passage (P16 + P23) HEK cells determined using a microplate reader. Torin1 autophagy induction is less potent in higher passage cells (EC_50_ = 97.6 nM) compared to low passage cells (EC_50_ = 15.9 nM), n = 4 per condition, 2 independent experiments. An extra sum-of-squares *F*-test (*F* (3, 90) = 13.29, *p* < 0.0001) revealed a significant difference between the best-fit values (Bottom, HillSlope, EC_50_) for each curve. Control values from the microplate reader measurements are tightly clustered around 100%, (100 ± 3.5%, mean ± SEM), hence a gray dashed line at 100% represents control autophagy, and fits were constrained to a top value of 100%.

The observations with AlloP led us to investigate factors that may affect sensitivity of the assay. During assay development, we observed a trend toward decreasing autophagy induction with Torin1 in successive experiments (Fig. 2a). We hypothesized that factors associated with cell division or age may modulate autophagic responses. Indeed, in a direct test of the hypothesis, we found that high-passaged cells (> passage 10, P10) showed an impaired Torin1 effect (Fig. 2b). The result of passage number was surprising given that HEK cells are immortalized cells^21^. Nevertheless, based on these results, we denoted < passage 10 as low-passage cells and > passage 10 as high-passage cells for subsequent experiments.

We moved next to a high-throughput, microplate reader format, allowing us rapidly to evaluate several NAS structures, concentrations, and interacting factors. We first used this format to investigate the effect of passage on Torin1 potency versus efficacy. Across independent replicates, passage increased the EC_50_ for Torin1 (Fig. 2c), thereby clarifying that passaging impairs potency of Torin1. Due to these observations, all subsequent experiments were conducted on low-passage cells < P10 to aid sensitivity of detecting autophagy induction.

### Little effect of NAS on autophagy in HEK cells; no substantial interaction with autophagy inducers

**Figure 3:**
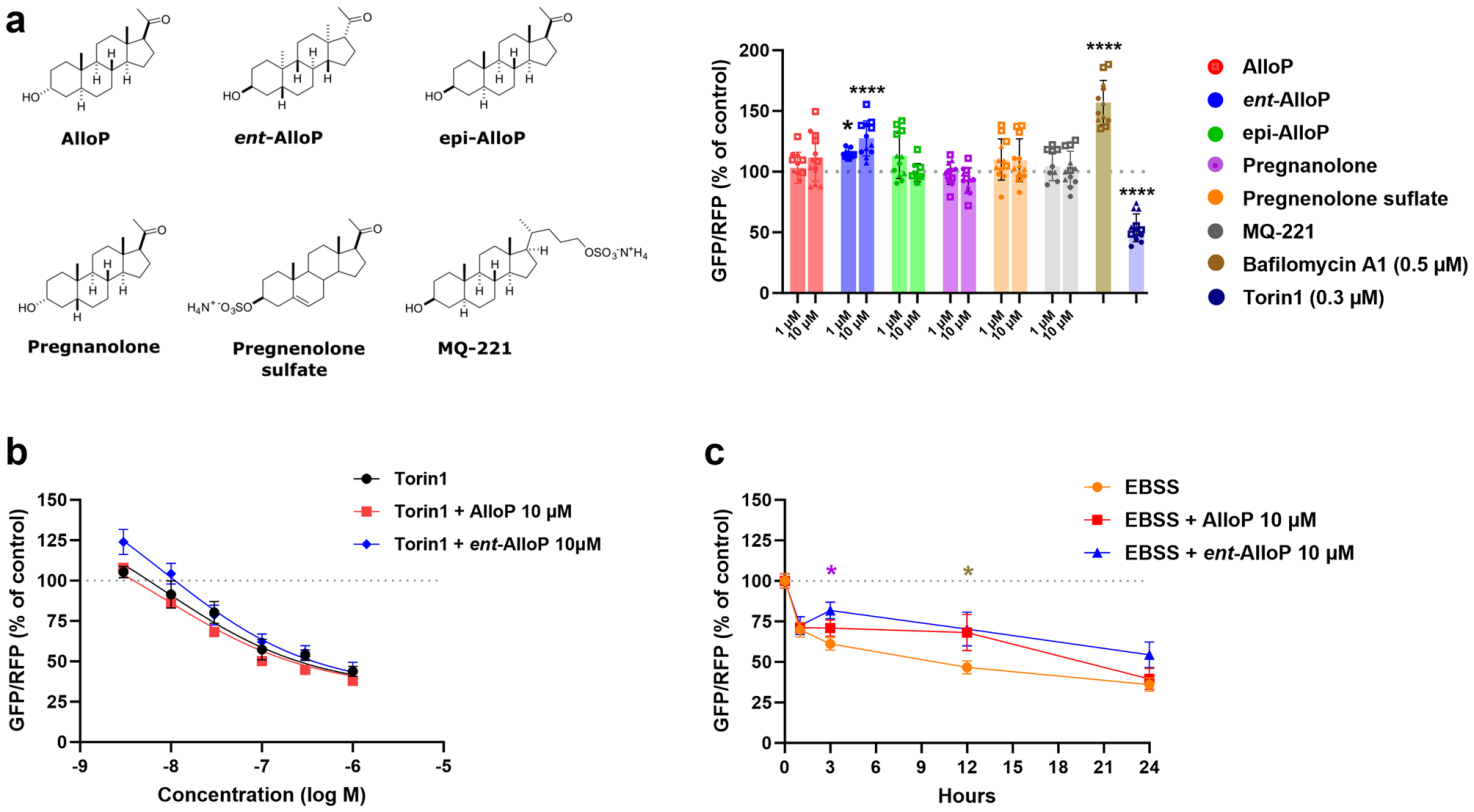
Little effect of NAS on autophagy or interaction with autophagy inducers. (**a**) A variety of structurally diverse NAS tested for their effects on autophagy at 1 and 10 µM, along with known autophagy inducer, Torin1 0.3 µM, and blocker, Bafilomycin_A1_ 0.5 µM. All treatments were for 24 h. Each point represents a well in a 96 well-plate (n = 4 replicates per condition from 3 separate experiments). Circles represent first experiment, squares second, and triangles third. Black asterisks represent significantly different conditions to the control based on results of separate one-way ANOVAs. 6 one-way ANOVAs (one per NAS) revealed an effect of *ent*-AlloP (*F* (2, 33) = 15.78, *p* < 0.0001). Dunnett’s multiple comparisons showed a difference between control and *ent*-AlloP 1 µM (*p = 0.0134) and *ent*-AlloP 10 µM (****p < 0.0001). However when accounting for multiple drug comparisons, two nested one-way ANOVAs (one for each concentration) revealed no differences from control for any NAS at a given concentration. Two unpaired t-tests, one of Torin1 and the other of Bafilomycin_A1,_ revealed significant effects of treatment (****p < 0.0001), including a Bonferroni correction for multiple comparisons. (**b**) Dose–response curves of Torin1, Torin1 + AlloP 10 μM, Torin1 + *ent*-AlloP 10 μM revealed a dose-dependent effect of Torin1 on autophagy induction and no statistical interaction of NAS with Torin1. An extra sum-of-squares *F*-test (*F* (6, 204) = 0.6823, *p* = 0.6641) revealed no difference between the best-fit values (Bottom, HillSlope, EC_50_) for each curve. The values of Torin1 0.3 µM are represented in (**a**) as a positive control since these treatments were conducted on the same plates. (**c**) Time course study of EBSS showed time-dependent autophagy. A two-way ANOVA revealed a difference between time (*F* (4, 165) = 33.92, *p* < 0.0001) and treatment (*F* (2, 165) = 5.712, *p* = 0.0040), with no significant interaction (*F* (8, 165) = 1.082, *p* = 0.3780). Dunnett’s multiple comparison showed a difference between EBSS and *ent*-AlloP at 3 h *p* = 0.0328 (purple asterisk), and EBSS and AlloP *p* = 0.0259 and *ent*-AlloP *p* = 0.0134 at 12 h (brown asterisk). In all panels of this figure, fluorescence was measured using a microplate reader, n = 4 per condition, 3 independent experiments.

We used the miniaturized microplate assay to re-evaluate AlloP and other NAS with diverse chemical structures. Although it is possible that the same NAS structural attributes important for positive actions at GABA-A receptors are required for effects on autophagy, we hypothesized that different structural attributes drive autophagy and that physiochemical properties are most important, for reasons described in the Introduction. Thus, we chose NAS with a variety of structures and charge status, with pregnenolone sulfate and MQ-221 bearing negative charge (Fig. 3a)^22,23^. Despite the weak effects of AlloP (Fig. 1), we suspected that uncharged NAS may be superior autophagy inducers due to their increased lysosomal penetration compared to charged NAS. We also expected a lack of enantioselectivity and stereoselectivity based on retinal results^14^. However, NAS with these diverse structural attributes produced no reliable autophagy modulation at 1 and 10 µM (Fig. 3a), despite the reliable performance of the positive control Torin1 (0.3 µM for 24 h). In fact, *ent*-AlloP may inhibit autophagy in this assay, based on one-way ANOVA analysis (Fig. 3a legend). To ensure there was no ceiling effect in our assay’s ability to detect autophagy inhibition, we treated cells with bafilomycin A_1_ (0.5 µM for 24 h; a potent autophagy inhibitor that blocks vacuolar H + ATPase) and observed robust GFP/RFP ratio increase relative to control, as expected (Fig. 3a). We next used a high-content imager, which allowed background subtraction, to evaluate AlloP effects. This high-sensitivity method revealed a strong effect of 500 nM Torin1 (GFP/RFP ratio 11.3 ± 1.4%) of control, but the AlloP (1 µM) effect was still negligible (101 ± 1.8% of control, mean ± SEM, n = 4 wells per condition from 1 experiment). The stronger Torin1 effect captured by the imager can be explained by background subtraction (non-background subtracted Torin1 effect, 84.5 ± 0.3% of control).

Because NAS induce autophagy in the presence of retinal stress^7^, we hypothesized that NAS may interact with known inducers of autophagy. We examined interaction with Torin1, rapamycin, and starvation using the high-throughput microplate reader. Although Torin1 (Fig. 3b) and starvation (Fig. 3c) produced dose and time dependent effects on autophagy, rapamycin did not strongly alter GFP/RFP ratio relative to control over a range of concentrations typically used in the literature (Supplementary Fig. 2A). Rapamycin’s weak effect was not necessarily a surprise, as others have found its efficacy to be cell-type dependent^24^. The weak effect persisted across two lots from the same supplier and a separate lot from a second manufacturer, with which we saw no changes in autophagy (Supplementary Fig. 2B). Torin1 produced robust changes in GFP/RFP ratio in a dose-dependent manner (Fig. 3b). Starvation (1, 3, 12, and 24 h) also produced robust changes in GFP/RFP ratio, reaching 50% of control by 12 h (Fig. 3b). In interaction tests, dose–response curves of Torin1 failed to be affected by either AlloP or *ent*-AlloP (Fig. 3b,c). With the non-pharmacological induction, we did observe unexpected increase of the starvation-induced GFP/RFP ratio relative to control at 3 h (*ent*-AlloP) and at 12 h (AlloP and *ent*-AlloP) (Fig. 3c).

### AlloP effect in astrocytes

**Figure 4:**
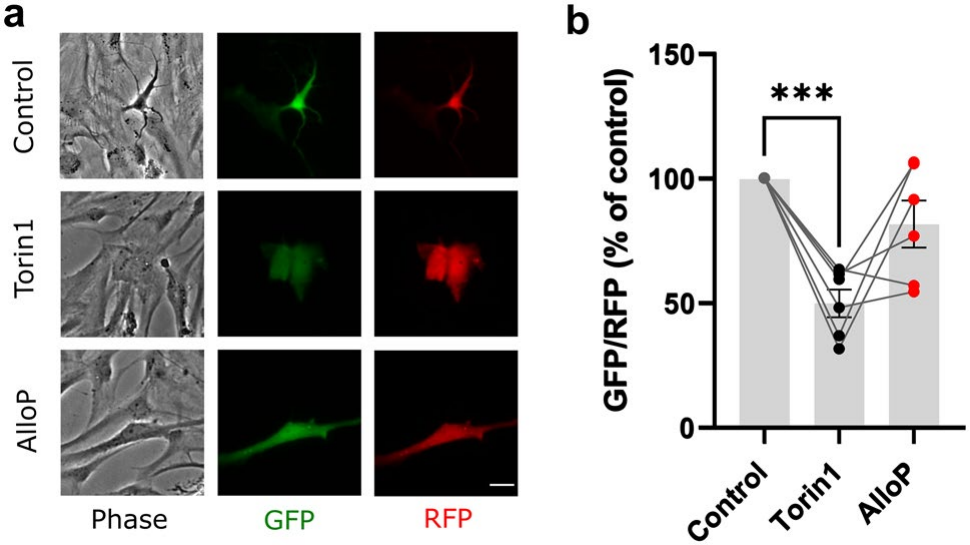
AlloP has little effect on autophagy in astrocytes. (**a**) Primary rat cortical astrocytes in phase contrast, GFP, and RFP channels at 10× after a 24 h treatment with 500 nM Torin1 and 10 µM AlloP. Scale bar: 25 µm. (**b**) Background-subtracted mean fluorescence ratios relative to their vehicle-treated control. Each normalized point represents a 35 mm dish (3–8 cells analyzed per dish; 6 independent experiments). Lines connect independent dishes treated during the same experiment. A one-way ANOVA (*F* (2, 15) = 15.98) showed a main effect of treatment, *p* = 0.0002, and Dunnett’s multiple comparisons revealed a difference between control and Torin1 (***p = 0.0001).

Apart from inducing autophagy in retinal ganglion cells, AlloP has been shown to induce autophagy in astrocytes^25^. To examine whether NAS effects are cell-type dependent, we transfected primary cortical astrocytes with the fluorescent probe. Transfected cells were verified as astrocytes with GFAP stain (Supplementary Fig. 3). We found that AlloP 10 µM did not reliably reduce GFP/RFP, although we cannot exclude a small effect, while Torin1 (500 nM) again robustly induced autophagy (Fig. 4).

## Discussion

Whether NAS with differing chemical structures differentially regulate autophagy is previously unexplored. Here, we attempted to adress this question by assaying several analogues with a fluorescent ratiometric probe. We first refined our assay to only use early passage cells (*P* < 10), as the potency of autophagy induction with Torin1 was dependent on cell passage. Despite optimizing sensitivity, contrary to expectations, we found that NAS failed to reliably increase autophagy in this cell model, including when tested for interaction in the context of known autophagy inducers Torin1 or EBSS starvation. Given evidence that AlloP induces autophagy in astrocytes^25^, we tested NAS in this cell type, but failed to show reliable modulation of autophagy. Taken together, we conclude that NAS effects on autophagy may be context and cell-type specfic. Although cell lines could in principle rapidly identify active analogues, consideration of cell-type appears to be important in the case of NAS and autophagy.

Passage effects on pharmacological autophagy induction were surprising, as HEK cells are an immortalized cell line and do not undergo senesence^21^. However, there is a large literature suggesting that autophagic activity declines with age in many organisms^26^. In neurons, this could be due to decreased lysosomal function, as cells in the hypothalamus from aged wild-type mice have less autolysosomal fusion and impaired delivery of autophagy substrates to lysosomes^27^. Factors associated with autophagic impairment during aging may be investigated in future experiments as potential contributors to the passage effects observed here. We also note that the cell based assay used here has advantages over other methods as described in the Introduction, particularly for our major goal of screening; however, for many experimenters Western blots remain the gold standard for autophagy assays. Although we had showed robust positive effects of Torin1, future work could extend the tests of passage effects and NAS to these standard assays of autophagy.

AlloP produced small but statistically significant changes to GFP/RFP ratios in microscopy experiments (Fig. 1), whereas reliable changes were not detected in the high-throughput microplate format (Fig. 2). One might expect that the contribution of many cells in the high-throughput format might drive lower sensitivity to small differences if cells respond heterogenously to drugs. It is also possible that the higher cell density of the high-throughput format is associated with differences in responsiveness. Perhaps lack of background subtraction in the plate reader experiments contributed to an inability to detect small changes to fluorescence ratios, but background-subtracted values from high-content imaging also produced a negligible AlloP effect. Both microscopy and the plate reader detected similar changes to fluorescence ratios of Torin1 (~ 50%), so it remains unclear that assay sensitivity can explain the differences in AlloP effects. Statistical significance aside, both assays suggest a weak biological impact of AlloP.

The context and cell type in which our experiments were executed may be crucial for reconciling our results with previous studies. Previously, AlloP protected rat retinal ganglion cells in a glaucoma model of elevated pressure, and protection was bafilomycin A1-sensitive^7^. Thus, autophagy appeared to mediate the neuroprotective effects of AlloP applied for 24 h at 1 μM, under a condition of high cellular stress that resulted in histological neuronal and axonal damage. Although we tried to replicate a stress-like environment in the HEK cell model withTorin1 and EBSS (Fig. 3b,c), we observed no reduction of the GFP/RFP ratio by NAS. In fact, both AlloP and *ent*-AlloP interfered with the effect of starvation at 12 h. This interference was consistent with trends toward increased fluorescence ratio observed with *ent*-AlloP alone (Fig. 3a). Perhaps the stress of ocular pressure (retinal studies) may induce signaling pathways other than those induced by Torin1 or starvation and be critical for activating the autophagic protective effects of AlloP. Further, because a key difference between our experiment and the previous study is cell type, NAS may interact with pathways specific to retinal ganglion cells to modulate autophagy with cell-type specificity.

Although we can exclude the possibility of a small effect, we also failed to observe strong AlloP induction of autophagy in primary rodent astrocytes (Fig. 4), contrary to an effect observed previously^25^. Although we used rat astrocytes instead of mouse and utilized slightly different growth medium, both studies used astrocytes derived from the cortex grown until 30 days in vitro. Our studies extended exposure from 1 h^25^ to 24 h and increased AlloP concentration from 250 nM (previous work) to 10 μM to in hopes of optimizing induction. We also used a ratiometric probe to assay autophagy, instead of immunoblots. Although sensitivity of the ratiometric probe used in our studies is an advantage^16^, it is possible that differences in the detection methods participate in the different outcomes. Overall, further work will be needed to reconcile these results, but our studies highlight the complexity inherent in replicating and extending even apparently straightforward results.

Rapamycin’s lack of dose-dependent effects on autophagy in HEK cells (Supplementary Fig. 2) could provide insight into lack of NAS effects. Others have found that rapamycin’s potency and efficacy are cell-type dependent^24^. This may result from high basal phosphatidic acid (PA) levels in HEK cells, which competes with rapamycin for a site on mTOR^28^. Low sensitivity may also result from the high stability of mTORC1 in these cells^24^. NAS may be subject to mitigating factors similar to rapamycin in HEK cells, despite likely different upstream mechanisms of action for initiating autophagy.

In summary, our work examines NAS effects on autophagy in HEK cells using a previously validated ratiometric fluorescent probe. We failed to find significant induction of autophagy of varied NAS structures at therapeutic and supratherapeutic concentrations, including when tested as allosteric modulators. Additionally, passage number of HEK cells influenced the potency of pharmacological autophagy induction; thus, passage should be carefully considered in experimental design. We conclude that context and cell-type hinder the ability to screen for autophagy induction. Future studies should carefully consider cell type to maximize relevance to neuropsychiatric treatment.

### Supplementary Information

Supplementary Figures.

## Data Availability

Data that support the findings of this study are available from the corresponding author on reasonable request.

## References

[1] Belmaker RH, Agam G (2009). Major depressive disorder. N. Engl. J. Med..

[2] Meltzer-Brody S, Colquhoun H, Riesenberg R, Epperson CN, Deligiannidis KM, Rubinow DR (2018). Brexanolone injection in post-partum depression: Two multicentre, double-blind, randomised, placebo-controlled, phase 3 trials. Lancet.

[3] Zorumski CF, Paul SM, Covey DF, Mennerick S (2019). Neurosteroids as novel antidepressants and anxiolytics: GABA-A receptors and beyond. Neurobiol. Stress.

[4] Gunduz-Bruce H, Silber C, Kaul I, Rothschild AJ, Riesenberg R, Sankoh AJ (2019). Trial of SAGE-217 in patients with major depressive disorder. N Engl J Med.

[5] Walkery A, Leader LD, Cooke E, Vandenberg A (2021). Review of allopregnanolone agonist therapy for the treatment of depressive disorders. Drug Des. Dev. Ther..

[6] Gassen NC, Rein T (2019). Is there a role of autophagy in depression and antidepressant action?. Front. Psychiatry.

[7] Ishikawa M, Takaseki S, Yoshitomi T, Covey DF, Zorumski CF, Izumi Y (2021). The neurosteroid allopregnanolone protects retinal neurons by effects on autophagy and GABRs/GABA_A_ receptors in rat glaucoma models. Autophagy.

[8] Gulbins A, Schumacher F, Becker KA, Wilker B, Soddemann M, Boldrin F (2018). Antidepressants act by inducing autophagy controlled by sphingomyelin–ceramide. Mol Psychiatry.

[9] Lieberman OJ, Frier MD, McGuirt AF, Griffey CJ, Rafikian E, Yang M (2020). Cell-type-specific regulation of neuronal intrinsic excitability by macroautophagy. Elife.

[10] Tomoda T, Sumitomo A, Shukla R, Hirota-Tsuyada Y, Miyachi H, Oh H (2021). BDNF controls GABA_A_R trafficking and related cognitive processes via autophagic regulation of p62. Neuropsychopharmacol.

[11] Kulkarni VV, Anand A, Herr JB, Miranda C, Vogel MC, Maday S (2021). Synaptic activity controls autophagic vacuole motility and function in dendrites. J. Cell Biol..

[12] Abdallah CG, Averill LA, Gueorguieva R, Goktas S, Purohit P, Ranganathan M (2020). Modulation of the antidepressant effects of ketamine by the mTORC1 inhibitor rapamycin. Neuropsychopharmacol.

[13] Covey DF, Evers AS, Mennerick S, Zorumski CF, Purdy RH (2001). Recent developments in structure–activity relationships for steroid modulators of GABA_A_ receptors. Brain Res. Rev..

[14] Ishikawa M, Nakazawa T, Kunikata H, Sato K, Yoshitomi T, Krishnan K (2022). The enantiomer of allopregnanolone prevents pressure-mediated retinal degeneration via autophagy. Front Pharmacol.

[15] Jiang X, Shu HJ, Krishnan K, Qian M, Taylor AA, Covey DF (2016). A clickable neurosteroid photolabel reveals selective Golgi compartmentalization with preferential impact on proximal inhibition. Neuropharmacology.

[16] Kaizuka T, Morishita H, Hama Y, Tsukamoto S, Matsui T, Toyota Y (2016). An autophagic flux probe that releases an internal control. Mol. Cell.

[17] Thomas P, Smart TG (2005). HEK293 cell line: a vehicle for the expression of recombinant proteins. J Pharmacol Toxicol Methods.

[18] Ghavami S, Shojaei S, Yeganeh B, Ande SR, Jangamreddy JR, Mehrpour M (2014). Autophagy and apoptosis dysfunction in neurodegenerative disorders. Prog. Neurobiol..

[19] Madureira M, Connor-Robson N, Wade-Martins R (2020). LRRK2: autophagy and lysosomal activity. Front. Neurosci..

[20] Ishikawa M, Yoshitomi T, Zorumski CF, Izumi Y (2014). Neurosteroids are endogenous neuroprotectants in an ex vivo glaucoma model. Invest. Ophthalmol. Vis. Sci..

[21] Graham FL, Smiley J, Russell WC, Nairn R (1977). Characteristics of a human cell line transformed by DNA from human adenovirus type 5. J. Gen. Virol..

[22] Park-Chung M, Malayev A, Purdy RH, Gibbs TT, Farb DH (1999). Sulfated and unsulfated steroids modulate gamma-aminobutyric acid_A_ receptor function through distinct sites. Brain Res..

[23] Ziolkowski L, Mordukhovich I, Chen DM, Chisari M, Shu HJ, Lambert PM (2021). A neuroactive steroid with a therapeutically interesting constellation of actions at GABA_A_ and NMDA receptors. Neuropharmacology.

[24] Nyfeler B, Bergman P, Triantafellow E, Wilson CJ, Zhu Y, Radetich B (2011). Relieving autophagy and 4EBP1 from rapamycin resistance. Mol. Cell. Biol..

[25] Kim HN, Lee SJ, Koh JY (2012). The neurosteroids, allopregnanolone and progesterone, induce autophagy in cultured astrocytes. Neurochem. Int..

[26] Aman Y, Schmauck-Medina T, Hansen M, Morimoto RI, Simon AK, Bjedov I (2021). Autophagy in healthy aging and disease. Nat Aging.

[27] Kaushik S, Arias E, Kwon H, Lopez NM, Athonvarangkul D, Sahu S (2012). Loss of autophagy in hypothalamic POMC neurons impairs lipolysis. EMBO Rep..

[28] Mukhopadhyay S, Frias MA, Chatterjee A, Yellen P, Foster DA (2016). The enigma of rapamycin dosage. Mol. Cancer Ther..

