# Supplementary material for: Neuroactive steroid effects on autophagy in a human embryonic kidney 293 (HEK) cell model
Source: Sci Rep. 2024 Jan 10;14:1042. doi: 10.1038/s41598-024-51582-x (PMC10781668; doi:10.1038/s41598-024-51582-x)
Supplement: Supplementary file 1 — Supplementary Figures. [file 41598_2024_51582_MOESM1_ESM.pdf]

Supplementary Information

Neuroactive steroid effects on autophagy in a human embryonic kidney 298 (HEK) cell model

Sofia V. Salvatore B.A.[1]; Ma. Xenia G. Ilagan Ph.D.[3]; Hongjin Shu Ph.D.[1]; Peter M. Lambert B.S.[1,4]; Ann Benz B.S.[1]; Mingxing Qian Ph.D.[2]; Douglas F. Covey Ph.D.[2,5]; Charles F. Zorumski M.D.[1,5]; Steven Mennerick Ph.D.[1,5]

Departments of Psychiatry[1] and Developmental Biology[2]
High-Throughput Screening Core, Center for Drug Discovery[3]
Medical Scientist Training Program[4]
Taylor Family Institute for Innovative Psychiatric Research[5]

Washington University in St. Louis School of Medicine
660 S. Euclid Ave., MSC 8134-0181-0G
St. Louis, MO 63110

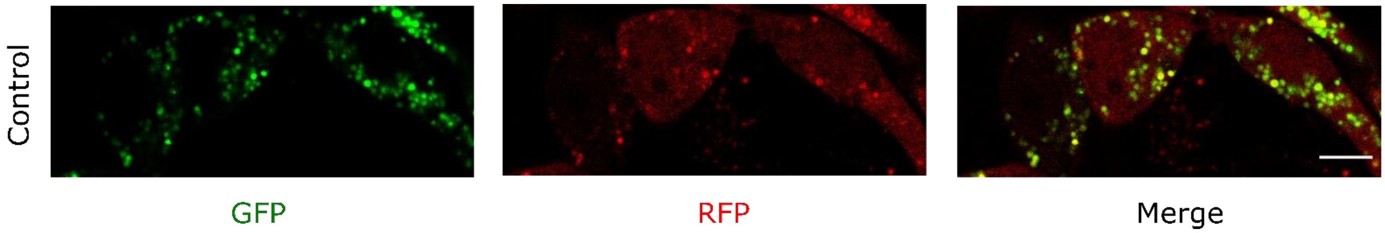

Supplementary Figure 1. GFP/RFP localization in transfected HEK cells.

HEK cells transfected with the pcDNA3-GFP-LC3-RFP-LC3ΔG probe at 60x show GFP and RFP puncta consistent with autophagosome localization. Scale bar: 10 μm.

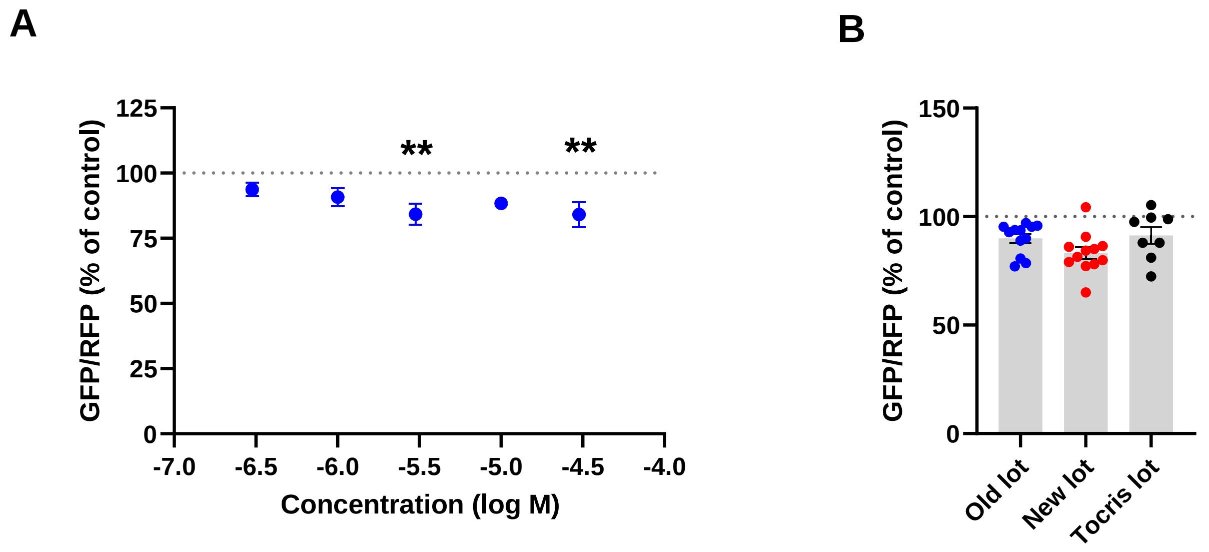

Supplementary Figure 2. HEK cells fail to respond to rapamycin in a dose-dependent manner.

(A) Lack of a dose response to rapamycin (nM to µM) in HEK cells analyzed on a microplate reader, n =4 per concentration, 6 independent experiments. A one-way ANOVA showed an effect of rapamycin relative to control ($F (5, 138) = 2.924$ $p = 0.0153$). Dunnett's multiple comparisons revealed a significant effect of 3 µM (**p = 0.0090) and 30 µM (**p = 0.0087), with 10 µM showing a trend towards significance ($p = 0.0853$). (**B)** A one-way ANOVA ($F (2, 29) = 2.556$ $p =0.0950$) revealed that the vendor source or lot of rapamycin did not appreciably alter the effect of 1 µM rapamycin for 24 h, n = 4 per condition, 3 independent experiments. Old lot was used in all rapamycin experiments for (A) and the new lot was from the same company as old, LC Laboratories.

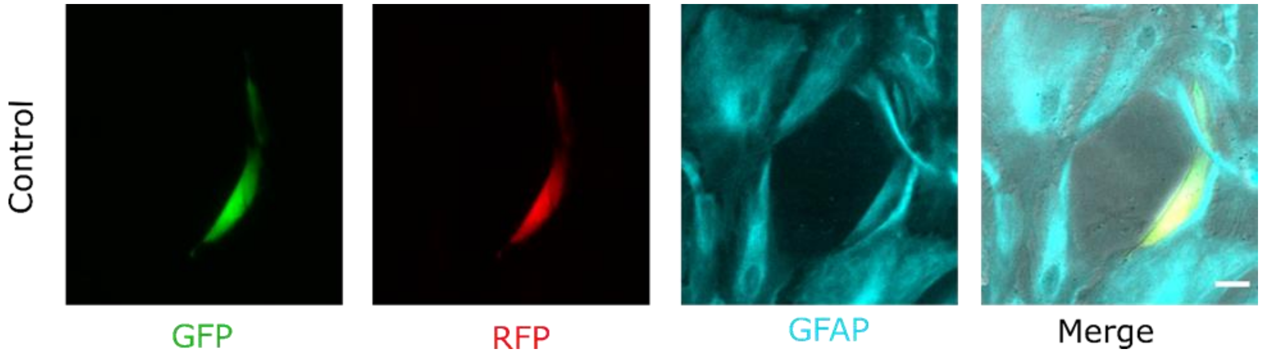

Supplementary Figure 3. Autophagy probe, GFP-LC3-RFP-LC3ΔG, is expressed in astrocytes.

Primary rat cortical astrocytes in GFP, RFP, and GFAP (immunostain) channels at 10x magnification reveal that transfected cells with GFP-LC3-RFP-LC3ΔG are astrocytes, as evidenced by the merged image. Scale bar: 25 μm.
